# Acute Aerobic Exercise Increases Cortical Activity during Working Memory: A Functional MRI Study in Female College Students

**DOI:** 10.1371/journal.pone.0099222

**Published:** 2014-06-09

**Authors:** Lin Li, Wei-Wei Men, Yu-Kai Chang, Ming-Xia Fan, Liu Ji, Gao-Xia Wei

**Affiliations:** 1 Key Laboratory of Adolescent Health Assessment and Exercise Intervention, Ministry of Education, Shanghai, China; 2 Physical Education and Health School, East China Normal University, Shanghai, China; 3 Shanghai Key Laboratory of Magnetic Resonance, East China Normal University, Shanghai, China; 4 Graduate Institute of Athletics and Coaching Science, National Taiwan Sport University, Taiwan; 5 Key Laboratory of Mental Health, Institute of Psychology, Chinese Academy of Sciences, Beijing, China; 6 Key Laboratory of Behavioral Science, Institute of Psychology, Chinese Academy of Sciences, Beijing, China; University of Florida, United States of America

## Abstract

There is increasing evidence that acute aerobic exercise is associated with improved cognitive function. However, neural correlates of its cognitive plasticity remain largely unknown. The present study examined the effect of a session of acute aerobic exercise on working memory task-evoked brain activity as well as task performance. A within-subjects design with a counterbalanced order was employed. Fifteen young female participants (M = 19.56, SD = 0.81) were scanned using functional magnetic resonance imaging while performing a working memory task, the N-back task, both following an acute exercise session with 20 minutes of moderate intensity and a control rest session. Although an acute session of exercise did not improve behavioral performance, we observed that it had a significant impact on brain activity during the 2-back condition of the N-back task. Specifically, acute exercise induced increased brain activation in the right middle prefrontal gyrus, the right lingual gyrus, and the left fusiform gyrus as well as deactivations in the anterior cingulate cortexes, the left inferior frontal gyrus, and the right paracentral lobule. Despite the lack of an effect on behavioral measures, significant changes after acute exercise with activation of the prefrontal and occipital cortexes and deactivation of the anterior cingulate cortexes and left frontal hemisphere reflect the improvement of executive control processes, indicating that acute exercise could benefit working memory at a macro-neural level. In addition to its effects on reversing recent obesity and disease trends, our results provide substantial evidence highlighting the importance of promoting physical activity across the lifespan to prevent or reverse cognitive and neural decline.

## Introduction

The effect of a single session of exercise, also known as acute exercise, on cognition has received growing interest since the pioneering review conducted by Tomporowski [1]. Although there have been some inconsistent findings, subsequent research has generally acknowledged an improvement of cognitive performance following exercise [2]. These facilitative effects of acute exercise on cognitive function have been further established by recent meta-analytical reviews [3], [4]. Despite being significantly positive, the magnitude of the detected impact (i.e., the effect sizes) of acute exercise on cognition has varied widely, suggesting that there are factors that moderate the relationship between acute exercise and cognitive function.

The type of cognitive function has been recognized as a primary moderator of exercise and cognition, most likely because exercise training leads, in particular, to more benefits on executive function relative to other cognitive categories (i.e., speed, spatial, and controlled) [5]. Executive function refers to a higher order of cognitive processes that supervise control over several lower levels of cognitive processes and guide the optimal behavior for achieving goal-directed behaviors [6]. These characteristics are responsible for the essential role that executive function plays in daily life. Remarkably, a disproportionate beneficial effect of exercise training has recently also been found for acute exercise. Specifically, although acute exercise improves multiple aspects of cognitive function, executive function receives the greatest positive effect, reflecting that acute exercise results in both general and specific improvements [7]. Notably, rather than acting as a unitary construct, executive function is composed of distinct sub-components, including working memory, inhibition, and shifting [8], and Etnier and Chang [9] postulated that acute exercise would have different influences on specific aspects of executive function. Nevertheless, the majority of studies addressing acute exercise have emphasized the inhibitory aspects of executive function [10], [11], leading to an insufficient overall understanding. Therefore, the present study attempted to examine other primary aspects of executive function, particularly working memory, to advance our understanding.

Working memory reflects an executive system involving limited resources that temporarily and simultaneously stores, maintains, and updates complex action-related information [12]. The maximum amount of information that an individual can retain, also known as the working memory capacity, is believed to serve a vital role underlying many cognitive processes and even other aspects of executive function [8]. Previous studies have indicated that working memory reacts sensitively to environmental changes, and improved performance has been observed after intensive cognitive training [13], [14]. However, only a few studies have investigated the impact of acute exercise on working memory, with inconsistent results. Specifically, these studies either found that acute exercise resulted in improvements in long-term memory, rather than working memory [15], or showed that exercise improved working memory, depending on the exercise modality (e.g., aerobic exercise) [16] and individual differences (e.g., individuals with a lower working memory) [17]. These few, but diverse studies make it difficult to formulate concrete hypotheses, and further investigation is needed.

In addition to behavioral measures, researchers utilize neuroimaging techniques to investigate the neural correlates underlying the improvement of cognition following acute exercise. Studies examining event-related potential (ERP) found that acute exercise is associated with an increased P300 amplitude and shortened P300 latency, indicating that acute exercise enhances attentional resource allocation as well as speeds the efficiency of classification for stimulus evaluation, thus providing the mechanism at the neural level [18], [19]. Such ERP studies have undoubtedly established a better understanding of the effects of acute exercise on cognition. However, ERP tends to be temporally oriented and provides limited information regarding the spatial locations of brain activity during certain cognitive tasks. Recently, functional magnetic resonance imaging (functional MRI) has been applied as a promising technique for observing changes in global cortical activity elicited by cognitive tasks [20], [21]. This technique measures the blood oxygenation that accompanies neuronal activity and establishes a bridge between cognitive function and brain activity, elucidating the neural systems related to exercise and cognition. Indeed, individuals with higher fitness show greater activation or more extensive neural networks in specific brain regions (e.g., the frontal and parietal cortices), which might, in turn, play a mediating role in the improvement of cognitive function. This technique can advance exercise-cognition research [20]–[22]. However, to our knowledge, studies investigating the effect of acute exercise on cognitive performance have yet to use functional MRI, and it remains largely unknown whether neural activity related to working memory is altered by acute exercise. Therefore, the use of functional MRI to explore the functional alterations of brain activity induced by acute exercise should receive more attention [23].

The present study aimed to investigate the effect of acute aerobic exercise on the working memory aspect of executive function while simultaneously investigating the underlying neural mechanism, as assessed through functional MRI. This technique was aimed at obtaining new insight linking exercise, executive function, and the brain. We hypothesized that acute aerobic exercise would improve working memory and change the functional pattern of brain activity associated with working memory.

## Materials and Methods

### Ethics Statement

The experimental procedure was approved by the Institutional Review Board of the East China Normal University and met the standards of the Declaration of Helsinki. Each participant was informed of the discomfort associated with acute exercise and provided written informed consent.

### Participants

Fifteen healthy female college students, aged 19 to 22 years, were voluntarily recruited through advertisements and fliers at East China Normal University. The sample size satisfied the criteria of a power analysis from a 2×4 mixed design with a power of 0.8, an alpha of 0.05, and an effect size of g = 1.41, where these parameters were based upon a recent meta-analysis that specifically focused on acute exercise and working memory [24]. The gender of the participants and their education level were restricted to avoid potential confounders of neural activation [25], [26]. The participants’ demographic characteristics were as follows: height (cm), M = 161.56, SD = 3.12; weight (kg), M = 50.75, SD = 3.44; body mass index, M = 19.45, SD = 1.38; and education (years), M = 14, SD = 0.00. All participants were first screened by the University’s Mental Counselling Center to ensure that they had no mental problems or disorders. Their medical and neurological histories were also screened, and individuals with a history of hearing or vision problems, physical injury, seizures, metal implants, head trauma with loss of consciousness, or substance abuse were excluded. The additional inclusion criteria were being right handed (Edinburgh Handedness Questionnaire), not currently menstruating (self-report question), and not pregnant. Importantly, participants were required to report their involvement in physical activities. As a result, none of the participants included in this study engaged in regular physical exercise. Furthermore, we requested that the participants get adequate sleep and not drink stimulating caffeine-containing beverages within 24 hours before the examination. In addition, we confirmed the usual MRI contraindications prior to functional MRI scanning (e.g., metallic objects or tattoos on the body).

### Working Memory Measures

A modified N-back task was programmed using E-Prime to assess working memory [27]. The N-back task consists of a sequence of stimuli, in which participants are asked to identify whether the current stimulus matches the stimulus from N steps earlier in the sequence, and the cognitive load is elevated with the operating number of N. The N-back task applied in the present study, designed using E-Prime software, consisted of 0-back, 1-back, and 2-back conditions, in which a letter stimulus (e.g., A to Z) was presented in a red, green, or yellow color, respectively. Each block condition included 18 trials (6 target trials and 12 non-target trials) in random order. Each letter stimulus was displayed on the screen for 2,000 ms. The participant was instructed to respond to the target trial by pressing a button on the keyboard with her right finger.

**Figure 1:**
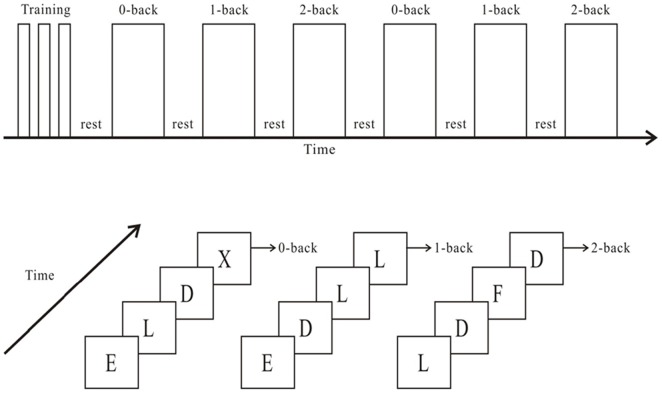
Schematic illustration of the experimental procedure and an example of the resultant stimulation under the three conditions of the N-back task.

In the 0-back condition, the target trial consisted of a stimulus presented as the letter “X”. In the 1-back condition, the target trial was a letter stimulus that was identical to the letter that immediately preceded it (i.e., the letter presented one trial back), whereas in the 2-back condition, the target trial was a letter stimulus that was identical to the letter presented two positions before it (i.e., the letter presented two trials back). The participants were asked to complete six alternating blocks, in the order 0-back, 1-back, and 2-back, twice, with 50-second resting intervals between each block (Figure 1). The total experiment duration was 422 seconds. Reaction time and accuracy were further identified as behavioral measures for each trial.

### Experimental Procedure

The present study employed a within-subjects design with a counterbalanced order to minimize the learning effect. The participants were tested individually in the laboratory and conducted the working memory task during functional MRI scans in two separate sessions, with a seven-day interval. The participants were also instructed to not undertake any physical activity or physical education course, and the sessions occurred at the same time of day at both visits.

During the first session, the participants were screened using the inclusion criteria, provided their demographic data, completed an ethical procedure, and went through a practice trial of the N-back task. The participants then underwent an acute session of aerobic exercise, including a 5-minute warm-up, cycling ergometer exercise for 20 minutes, and a 5-minute cool-down. The intensity of exercise was set at a heart rate of 120 beats/min on a cycling ergometer, with a graded work load of a 60–70 rpm/min pedaling rate. The work rate of the acute exercise, corresponding to 60% to 70% of the estimated maximal heart rate (calculated based on 220-age), has been shown to improve multiple aspects of cognitive function [18], [28]. Following the acute exercise session, the participants were instructed to conduct the N-back task while undergoing a functional MRI scan within 15 minutes after the cessation of exercise.

The second session was a control session that consisted of 20 minutes of quiet, seated resting. The participants were then scanned while performing the N-back task again, in an manner identical to the first session. Their heart rates were assessed with a heart rate monitor (Suunto t6, Serial No. 60502677, Finland) with recordings set to occur at 10-second intervals throughout the experimental process. At the end of the experimental protocol, the participants were briefly informed of the purpose of the research.

### Functional MRI Data Acquisition and Scanning Protocol

All brain images were acquired using a 3T Trio Tim scanner (Siemens, Erlangen, Germany) with a 32-channel head matrix coil. The N-back task was presented with ESys by Invivo Company. Functional images were obtained using an echo-planar imaging (EPI) sequence, with the following scan parameters: TR = 2,000 ms, TE = 30 ms, flip angle (FA)  = 90°, slice thickness  = 3.75 mm, field of view (FOV)  = 192×192 mm^2^, and voxel size  = 3.0×3.0×3.75 mm^3^. The resulting data included 243 brain volumes with 32 axial slices. During the functional MRI scans, all of the subjects were instructed to keep their eyes closed, relax, and move as little as possible. High-resolution structural images were acquired using a magnetization-prepared rapid gradient echo (MPRAGE) three-dimensional T1-weighted sequence (TR = 2530 ms, TE = 2.34 ms, T1 = 1100 ms, FA = 7°, thickness  = 1 mm, field of view (FOV)  = 256×256 mm^2^, voxel size  = 1.0×1.0×1.0 mm^3^).

### Statistical Analysis of Functional MRI Data

Functional image preprocessing and statistical analyses were carried out using SPM8 (http://www.fil.ion.ucl.ac.uk/spm/software/spm8/). The first five images of each time series were excluded to account for T1-stabilization effects. For each participant, EPI images were slice-time corrected and realigned to the first image, followed by normalization to the standard Montreal Neurological Institute EPI template and spatial smoothing using a Gaussian kernel (8 mm^3^ full width at half maximum). Contrast images were generated for each participant for the contrasts of interest (0-back > rest, 1-back > rest, 2-back > rest, respectively, in the control and exercise sessions), representing the pair-wise comparison of parameter estimates for the conditions, and then, separate single-group *t* tests were used to identify within-group contrasts of interest. Differences between any of the two groups were examined using two-sample *t* tests, masked by the single-sample *t* test results. All statistical maps were set at a level of an uncorrected cluster significance threshold of *p* = 0.001 (Z>2.3) and a cluster size >5 voxels [29]–[31]. Activation maps were overlaid on the group mean high-resolution images for display purposes.

## Results

### Behavioral Performance

Table 1 presents the detailed behavioral measures obtained based on the treatment session and the N-back condition. A 2 (session: control versus exercise) ×3 (N-back task condition: 0-back, 1-back, versus 2-back) repeated-measures ANOVA of reaction time revealed a significant main effect of the N-back task condition (*F* (2, 28)  = 8.18, *p*<.01, partial eta square  = .37). A follow-up post-hoc analysis revealed that the reaction time under the 2-back condition was significantly longer than that under the 1-back and 0-back conditions. However, no significant differences were found for the main effect of session or the interaction of the session and condition (*p*>.05).

**Table 1:** Behavioral performances under the N-back task conditions in the control and exercise sessions.

	Control session		Exercise session	
Condition	Accuracy (%)	Reaction Time (ms)	Accuracy (%)	Reaction Time (ms)
0-back	95.15±4.13	310.51±22.56	94.09±5.32	304.34±25.68
1-back	92.58±6.26	315.54±21.22	92.62±5.62	312.66±19.14
2-back	84.64±6.19	326.69±19.03	87.96±6.53	318.97±22.01

Similar to the results for the reaction time, a 2×3 ANOVA of accuracy revealed that there was a significant main effect of the N-back task condition (*F* (2, 28)  = 16.40, *p*<.01, partial eta square  = .54). Follow-up post-hoc analysis revealed that the accuracy under the 2-back was significantly lower than that under the 1-back and 0-back conditions. No significant differences were found for the main effect of session or the interaction of the session and condition (*p*>.05).

Moreover, the mean heart rate was 83.23±6.36 beats per minute (bpm) in the resting state and 135.37±16.43 bpm during exercise.

### Functional MRI Results

**Figure 2:**
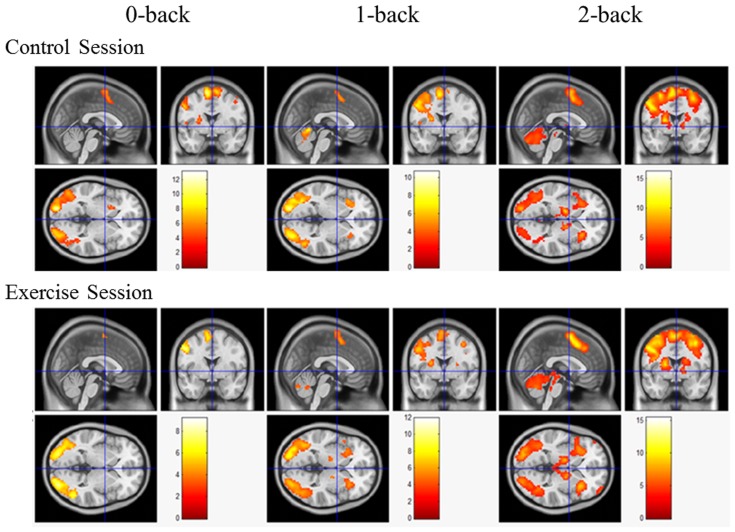
Brain activation maps during the performance of the N-back task in the exercise session and the control session at the level of *p*<0.001 (uncorrected), Z>2.3, and a cluster size >5 voxels for comparisons using cluster detection. Red areas indicate greater activation during the N-back condition relative to the resting condition.

A main effect of the N-back condition was generally observed in both the control and exercise sessions. The functional MRI results revealed that the 2-back condition was associated with the most extensive activation of brain regions (i.e., the frontal lobe, subcortical area, occipital lobe, parietal lobe, and temporal lobe), whereas activation in the 0-back condition was associated with the smallest number of brain regions (e.g., the frontal lobe and occipital lobe) in the exercise session (Figure 2, also see Table S1). Similarly, the regional brain activation in the 2-back condition was the most extensive (i.e., frontal lobe, subcortical area, occipital lobe, parietal lobe, and temporal lobe), whereas the 0-back condition induced activation of the smallest number of brain regions (e.g., the frontal lobe, subcortical area, occipital lobe, and temporal lobe) in the control session (see Figure 2 and Table S1).

Additional analysis of the brain regions activated under the three N-back conditions revealed that the commonly activated regions were the left precentral gyrus and the left inferior occipital lobe in the exercise session versus the left precentral gyrus, the right cerebellum, and the supplementary motor areas in the control session. Among these regions, the cluster sizes generally corresponded to the cognitive load, with the largest cluster size being found under the 2-back condition and the smallest cluster size under the 0-back condition (Table 2).

**Table 2:** Cluster sizes of commonly activated brain regions under the N-back task conditions in the control and exercise sessions.

		N-back Conditions		
		0-back	1-back	2-back
Control session				
	Left precentral gyrus	22	68	1031
	Right cerebellum	31	49	584
	Supplementary motor area	163	65	5
Exercise session				
	Left precentral gyrus	33	58	2034
	Left Inferior Occipital lobe	226	391	342

**Figure 3:**
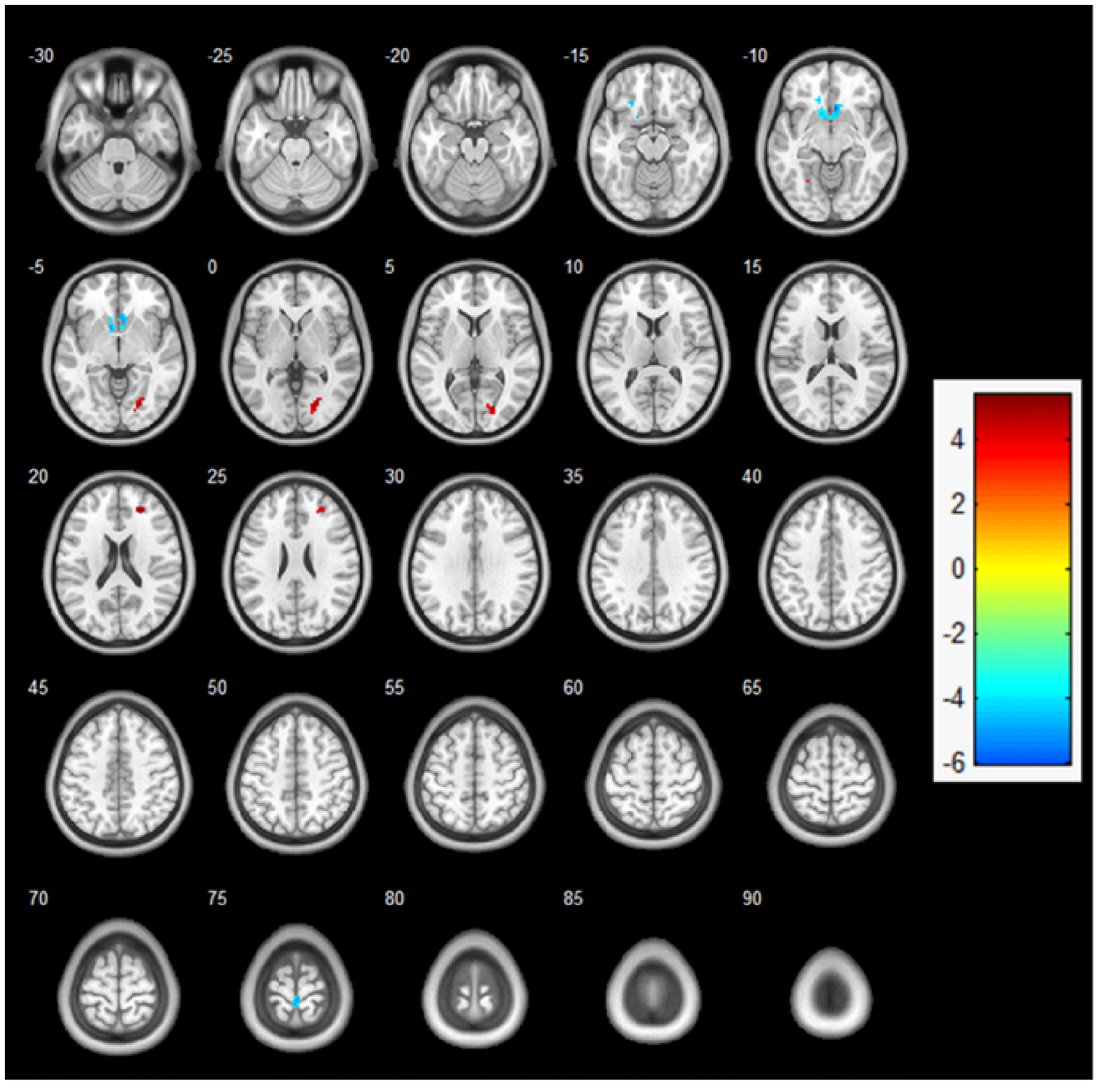
Two-sample t-test analysis: fMRI activation maps under the 2-back condition between the exercise and control sessions (exercise session > control session) at the level of *p*<0.001 (uncorrected), Z>2.3, and a cluster size >5 voxels for comparisons using cluster detection. Red areas indicate greater activation during the 2-back condition in the exercise session relative to the control session. Blue areas indicate greater activation during the 2-back condition in the control session relative to the exercise session.

Regarding contrasts between the exercise and control sessions, a two-sample t-test revealed a significant difference in activation between the exercise and control sessions under the 2-back condition (*p*<0.001, uncorrected), whereas no significant differences were observed between the two sessions under the 1-back and 0-back conditions (Figure 2). Specifically, the acute exercise session resulted in greater activation of the right middle frontal gyrus (BA 10), the right lingual gyrus (BA 17), and the left fusiform gyrus (BA 19) and less activation of the anterior cingulate cortex (BA 32), the left inferior frontal gyrus (BA 47), and the right paracentral lobule (BA 4) relative to the control session under the 2-back condition (Figure 3, Table 3).

**Table 3:** Brain activation comparisons during the 2-back task in the exercise and control sessions.

Brain region (Brodmann, BA)	Cluster Size	MNI coordinates			Z score	t	p
		X	Y	Z			
Exercise > Control							
Right middle frontal gyrus (BA 10)	19	24	45	21	5.03	3.74	0.000
Right lingual gyrus (BA 17)	50	21	−78	3	5.33	3.88	0.000
Left fusiform gyrus (BA 19)	6	−27	−57	−12	4.12	3.28	0.000
Control > Exercise							
Anterior cingulate cortex (BA 32)	57	6	21	−9	5.92	4.12	0.000
Right paracentral lobule (BA 4)	20	3	−33	75	5.08	3.76	0.000
Left inferior frontal gyrus (BA 47)	17	−15	30	−12	4.82	3.64	0.000

## Discussion

A rapidly growing body of literature indicates that acute aerobic exercise improves cognitive function from both the behavioral and neuroelectric perspectives. However, no study had previously investigated the effect of acute exercise on working memory using functional MRI. To our knowledge, the present study is the first designed to examine the behavioral change of working memory assessed in an N-back task following the cessation of acute exercise and to explore the macro-scale level of the neural system underlying the effect of exercise on working memory. The results are partially consistent with our prediction; although moderate acute exercise failed to improve working memory at the behavioral level, acute exercise significantly influenced brain activation under the 2-back condition, with increased activation of the right middle prefrontal gyrus, the right lingual gyrus, and the left fusiform gyrus and decreased activation of the anterior cingulate cortexes, the left inferior frontal gyrus, and the right paracentral lobule. These results may be of great importance for understanding the neural correlates of the effect of acute exercise on working memory.

The lack of significant differences in behavioral performance found between the exercise and control sessions contradicted our hypothesis. The failure to replicate previous findings may be attributed to the small sample size, as previous studies included sample sizes ranging from 20 to 48 [16], [17]. However, the absence of an effect of acute exercise on working memory is not unique to this study. In a study involving a similar number of participants [15], acute exercise only positively impacted long-term memory, whereas there was no improvement in the cognitive task applied to measure working memory. Similarly, a recent study indicated that an acute session of yoga (but not aerobic exercise on a treadmill) improved working memory performance, suggesting that an exercise mode other than aerobic exercise may benefit working memory [32]. Clearly, further research will be required to replicate these findings by increasing sample sizes and examining different types of exercise.

The present study showed that brain activation corresponded to the applied N-back conditions, regardless of the treatment session. Specifically, the 2-back condition resulted in global brain activation, including the frontal lobe, occipital lobe, parietal lobe, temporal lobe, and subcortical area, whereas the 0-back condition activated limited brain regions, such as the frontal and occipital lobes. Additionally, the resultant cluster sizes were generally observed to be larger under the 2-back condition and smallest under the 0-back condition, implying that an increased load of executive control was positively correlated with extensive activated brain regions and with larger cluster sizes. The finding that brain activation reflected the demand for executive control is in agreement with previous studies [33], [34] and thus confirms the validity of the applied executive function task and functional MRI modality.

Functional MRI scanning identified differential activation patterns in several brain regions under the 2-back condition in response to exercise relative to the control session. Specifically, acute exercise was positively associated with regional activation in the prefrontal lobe (i.e., the right middle frontal gyrus) and occipital lobe (including the right lingual gyrus and left fusiform gyrus). The prefrontal cortex is considered the brain region that is typically activated during the performance of a working memory task. The right middle frontal gyrus, located in the ventral and frontal pole area, is believed to reflect the coordination and shifting of information among multiple cognitive tasks and functions as an important area for executing and regulating working memory [33], [35]. Similarly, the detection of activation in both the lingual and fusiform areas located in the medial occipital cortex distinguished tasks involving different levels of working memory, suggesting that the solving of complex tasks can be attributed to these brain regions [36]. Our finding that acute exercise is associated with these brain regions is consistent not only with our hypothesis but also with recent studies using functional near-infrared spectroscopy (NIRS) [37], [38]. Tsujii, Komatsu and Sakatani [38] indicated that moderate acute aerobic exercise increases both working memory and cortical hemodynamic responses in the prefrontal cortex. With an emphasis on other executive controls (e.g., interference), similar results were also observed, including improved behavioral performance following a short session of aerobic exercise, with the right frontopolar area specifically being activated [37]. These findings suggest that these specific brain cortexes play vital roles in the alteration of executive function induced by acute exercise. The present study also found that these specific regions following acute exercise were markedly activated under the 2-back condition, which involved a higher executive control load, but not under the 1-back or 0-back condition. This result suggests that acute exercise may have greater effect on an executive function task that required a heavier cognitive load. These disproportional effects of acute exercise are in agreement with previous studies using neuroelectric techniques [18], [39]. Along with the robust relationship between the prefrontal cortex and executive function, our study extends knowledge regarding the relationship between acute exercise and neuronal activation by providing a spatial perspective at a macro-level using the functional MRI technique.

Interestingly, we also observed deactivation in the anterior cingulate cortexes and the left inferior frontal gyrus following the termination of acute exercise, which may provide an alternative viewpoint to explain the beneficial effect of acute exercise. The anterior cingulate cortex, located in the medial frontal lobe, is believed to reflect the response conflict and adaptation in monitoring the attentional control network [40]. To enhance cognitive processes, reduction of activity in the anterior cingulate cortex occurs during goal-directed cognitive demand [41]. These decreases in brain activation constitute the “default mode of brain function” hypothesis [41], [42]. The deactivation in the anterior cingulate cortex observed in the present study agrees with the default mode hypothesis and provides another interpretation of the facilitative effect of acute exercise on cognition at the macro-scale level of the brain. Moreover, the left inferior frontal gyrus, located in the ventral lateral prefrontal gyrus, is thought to play an executive role in semantic processing, including encoding and retrieval processing [43]. Given that acute exercise activated the right frontal gyrus and deactivated the left frontal gyrus simultaneously (implying an advantage of the specific frontal hemisphere), the results of the present study possibly indicate that decreased activation in the left hemisphere might represent a reduction of semantic processing or may refer to functional compensation for the increased activation of the contralateral hemisphere. This hypothesis warrants further examination.

Factors limiting our interpretation should be acknowledged, and further investigation is required. The findings of the present work may be restricted by the small sample size, as neuroimaging requires substantial statistical power. Further research with a larger sample size is encouraged; however, the sample examined in the present study could accurately reflect brain activity in accordance with the complexity of the cognitive task and differences in brain activation induced by acute exercise, suggesting that acute exercise does have a positive impact on brain activation. Another limitation could be the gender of participants in the present work. Because our participants were restricted to a specific gender (female) and because this restriction could potentially be a confounding factor, generalization of the current results should be carried out cautiously. However, previous studies investigating acute exercise and cognition have not examined the role of gender or whether gender moderates this relationship. Future research should employ a larger sample size with a proper gender distribution. Finally, future studies should monitor the physiological state or the mental state with objective measurement during scanning to exclude the influence of other potential factors on the results.

In conclusion, the results of the present study indicated that moderate acute aerobic exercise had a limited influence on working memory. Nevertheless, acute exercise activated the prefrontal and occipital cortexes, which are associated with executive control, particularly in the task involving conditions incurring greater cognitive demand. A similar positive influence of acute exercise was also revealed by the deactivation of the anterior cingulate cortex and left frontal hemisphere, implying that acute exercise leads to a default mode status and functional compensation during the performance of complex executive processes. Based on these preliminary positive findings, future research on macro-scale neural network or functional connectivity is warranted to explore the relationships among acute exercise, executive function, and the brain using a functional MRI approach to advance our understanding.

## Supporting Information

Table S1 Brain activation of N-back tasks and treatment sessions. It showed the cluster size, coordinates and peak intensity for each activated brain region under 6 conditions (sessions* N-back task).
(DOCX)
