# Supplementary material for: Acute Aerobic Exercise Increases Cortical Activity during Working Memory: A Functional MRI Study in Female College Students
Source: PLoS One. 2014 Jun 9;9(6):e99222. doi: 10.1371/journal.pone.0099222 (PMC4050105; doi:10.1371/journal.pone.0099222)
Supplement: Table S1 — Brain activation of N-back tasks and treatment sessions. It showed the cluster size, coordinates and peak intensity for each activated brain region under 6 conditions (sessions* N-back task). (DOCX) [file pone.0099222.s001.docx]

Table S1 Brain activation to N-back tasks and treatment sessions

| Brain region (Brodmann, BA) | Cluster Size | MNI coordinates | | | Peak  Intensity |
| --- | --- | --- | --- | --- | --- |
|  |  | X | Y | Z |  |
| 0-back in control session |  |  |  |  |  |
| Frontal Lobe |  |  |  |  |  |
| Precentral_L (BA 6) | 22 | -54 | 0 | 39 | 7.71 |
| Supp_Motor_Area_L | 163 | -9 | -3 | 66 | 11.04 |
| Subcortical Area |  |  |  |  |  |
| Cerebelum_6_R (aal) | 31 | 39 | -48 | -30 | 6.24 |
| Putamen_L | 8 | -24 | 6 | 12 | 6.82 |
| Occipital Lobe |  |  |  |  |  |
| Occipital_Mid_L | 389 | -21 | -93 | 0 | 13.09 |
| Temporal Lobe |  |  |  |  |  |
| Inferior Temporal Gyrus | 210 | 48 | -69 | -6 | 8.46 |
| Temporal_Sup_L | 12 | -54 | -42 | 21 | 6.76 |
|  |  |  |  |  |  |
| 1-back in control session |  |  |  |  |  |
| Frontal Lobe |  |  |  |  |  |
| Precentral_L (BA 9) | 68 | -51 | 3 | 39 | 7.49 |
| Supp_Motor_Area_L (BA 6) | 65 | -9 | 6 | 57 | 10.68 |
| Subcortical Area |  |  |  |  |  |
| Cerebelum_6_R (aal)/Culmen | 49 | 33 | -48 | -30 | 7.60 |
| Cerebelum_Crus1_L (aal)/Declive | 11 | -9 | -75 | -24 | 6.78 |
| Cerebelum_6_R (aal)/Declive | 10 | 9 | -72 | -21 | 6.23 |
| Vermis_4_5 (aal)/Culmen | 22 | 0 | -54 | -6 | 7.61 |
| Occipital Lobe |  |  |  |  |  |
| Lingual Gyrus | 188 | -21 | -90 | 0 | 9.53 |
| Cuneus (BA 17) | 227 | 15 | -96 | -6 | 9.58 |
| Parietal Lobe |  |  |  |  |  |
| Parietal_Inf_L (aal) | 144 | -27 | -57 | 48 | 8.45 |
| Temporal Lobe |  |  |  |  |  |
| Fusiform Gyrus (BA 37) | 54 | -48 | -60 | -15 | 7.23 |
|  |  |  |  |  |  |
| 2-back in control session |  |  |  |  |  |
| Frontal Lobe |  |  |  |  |  |
| Frontal_Mid_L (BA 10) | 12 | -42 | 57 | 12 | 6.72 |
| Frontal_Inf_Oper_R | 37 | 63 | 15 | 21 | 9.14 |
| Middle Frontal Gyrus (BA 10) | 9 | -36 | 57 | 24 | 6.71 |
| Precentral_L (BA 8) | 1031 | -54 | 6 | 42 | 13.39 |
| Precentral_R (BA 8) | 27 | 54 | 12 | 45 | 6.61 |
| Frontal_Mid_R | 195 | 33 | -3 | 57 | 10.20 |
| Supp_Motor_Area_L (BA 6) | 5 | -12 | -12 | 72 | 6.17 |
| Subcortical Area |  |  |  |  |  |
| Right Cerebellum/Declive | 584 | 27 | -57 | -36 | 12.55 |
| Cerebelum_6_L (aal)/Culmen | 170 | -30 | -54 | -30 | 10.88 |
| Right Brainstem | 9 | 6 | -27 | -6 | 6.52 |
| Thalamus_R | 24 | 15 | -6 | 0 | 7.72 |
| Putamen_L | 251 | -21 | 3 | 12 | 11.89 |
| Insula_R | 87 | 30 | 27 | 3 | 9.05 |
| Extra-Nuclear | 15 | 21 | 9 | 12 | 7.02 |
| Occipital Lobe |  |  |  |  |  |
| Occipital_Mid_L | 49 | -21 | -93 | 3 | 8.64 |
| Parietal Lobe |  |  |  |  |  |
| Parietal_Inf_R | 502 | 33 | -45 | 45 | 11.56 |
| Parietal_Inf_L (BA 40) | 672 | -42 | -45 | 48 | 16.21 |
| Temporal Lobe |  |  |  |  |  |
| Temporal_Inf_L | 216 | -48 | -60 | -12 | 9.83 |
|  |  |  |  |  |  |
| 0-back in exercise session |  |  |  |  |  |
| Frontal Lobe |  |  |  |  |  |
| Precentral_L (BA 6) | 33 | -57 | 3 | 42 | 9.21 |
| Supp_Motor_Area_L (BA 24) | 15 | -9 | 6 | 51 | 7.06 |
| Occipital Lobe |  |  |  |  |  |
| Occipital_Inf_L | 226 | -42 | -78 | -6 | 8.80 |
| Occipital_Inf_R | 209 | 42 | -78 | -6 | 8.03 |
|  |  |  |  |  |  |
| 1-back in exercise session |  |  |  |  |  |
| Frontal Lobe |  |  |  |  |  |
| Frontal_Inf_Tri_R | 14 | 33 | 27 | 9 | 6.40 |
| Precentral_L (BA 9) | 58 | -48 | 6 | 39 | 11.11 |
| Supp_Motor_Area_L | 49 | -9 | 9 | 57 | 8.46 |
| Subcortial Area |  |  |  |  |  |
| Cerebelum_6_R (aal)/Declive | 10 | 30 | -60 | -30 | 6.35 |
| Putamen_L | 8 | -24 | 0 | 15 | 6.12 |
| Occipital Lobe |  |  |  |  |  |
| Occipital_Inf_R (BA 19) | 334 | 42 | -78 | -9 | 9.21 |
| Occipital_Inf_L | 391 | -36 | -87 | -6 | 11.93 |
| Parietal Lobe |  |  |  |  |  |
| Parietal_Inf_L | 30 | -27 | -54 | 42 | 6.56 |
|  |  |  |  |  |  |
| 2-back in exercise session |  |  |  |  |  |
| Frontal Lobe |  |  |  |  |  |
| Frontal_Inf_Tri_R (BA 13) | 150 | 36 | 21 | 9 | 9.44 |
| Precentral_L | 2034 | -54 | 3 | 42 | 15.39 |
| Middle Frontal Gyrus (BA 10) | 9 | 39 | 63 | 6 | 6.88 |
| Middle Frontal Gyrus (BA 46) | 36 | -45 | 54 | 97.7 | 7.73 |
| Frontal_Inf_Oper_R | 19 | 51 | 12 | 18 | 6.45 |
| Frontal_Mid_R (BA 9) | 90 | 39 | 33 | 33 | 9.68 |
| Subcortical Area |  |  |  |  |  |
| Left Cerebellum | 135 | -33 | -60 | -30 | 8.27 |
| Right Cerebellum | 110 | 33 | -57 | -30 | 8.42 |
| Right Cerebellum | 118 | 12 | -75 | -27 | 8.12 |
| Right Brainstem | 16 | 6 | -21 | -9 | 7.00 |
| Lingual_R | 111 | 24 | -90 | -3 | 7.52 |
| Left Brainstem | 23 | -6 | -24 | -6 | 6.74 |
| Sub-Gyral | 16 | 39 | -57 | 0 | 6.76 |
| Sub-lobar | 132 | -21 | -3 | 15 | 9.59 |
| Medial Globus Pallidus | 22 | 18 | -6 | 0 | 8.02 |
| Caudate_R | 11 | 18 | -3 | 21 | 6.32 |
| Occipital Lobe |  |  |  |  |  |
| Occipital_Mid_L | 342 | -36 | -84 | -3 | 9.03 |
| Parietal Lobe |  |  |  |  |  |
| Parietal_Inf_L | 576 | -27 | -54 | 42 | 13.46 |
| Parietal_Inf_R (BA 40) | 370 | 39 | -51 | 51 | 8.80 |
| Temporal Lobe |  |  |  |  |  |
| Temporal_Inf_R | 13 | 57 | -54 | -9 | 6.42 |

L = left; R = right; Inf = inferior; Sup = superior; Mid = middle
